# Effects of Olopatadine Hydrochloride, a Histamine H_**1**_ Receptor Antagonist, on Histamine-Induced Skin Responses

**DOI:** 10.1155/2010/638051

**Published:** 2010-09-16

**Authors:** Takashi Hashimoto, Norito Ishii, Takahiro Hamada, Teruki Dainichi, Tadashi Karashima, Takekuni Nakama, Shinichiro Yasumoto

**Affiliations:** Department of Dermatology, Kurume University School of Medicine and Kurume University Institute of Cutaneous Cell Biology, 67 Asahimachi, Kurume, Fukuoka 830-0011, Japan

## Abstract

Effects of olopatadine hydrochloride, a histamine H_1_ receptor antagonist, on histamine-induced skin responses were evaluated in 10 healthy subjects in comparison with placebo, fexofenadine hydrochloride, and bepotastine besilate. Olopatadine significantly suppressed histamine-induced wheal, flare, and itch, starting 30 minutes after oral administration. Olopatadine was more effective than fexofenadine and bepotastine. None of the drugs studied impaired performance of word processing tasks. These results suggest that olopatadine can suppress skin symptoms caused by histamine soon after administration.

## 1. Introduction

Urticaria is a skin disease which is induced by chemical mediators, mainly histamine, released from skin mast cells by some stimulation. These chemical mediators produce flare and wheal and induce itch by stimulating sensory nerves [1]. Oral histamine H_1_ receptor antagonists (antihistamines) are the first line of treatment for idiopathic urticaria and other types of urticaria. Second-generation antihistamines are commonly used, because they show lower central nervous system depression and anticholinergic effects than first-generation drugs. Efficacy, rapidness and duration of action, and side effects, such as sleepiness and sedation, are known to vary from drug to drug. Itch affects patient's quality of life. Therefore, according to a questionnaire survey, urticaria patients prefer oral therapy that effectively and promptly relieves distressing itch [2]. The patients also prefer the drug with lower side effects.

In this paper, we present a clinical and pharmacological study of histamine-induced skin response test using iontophoresis on two major concerns of urticaria patients, that is, efficacy and side effects of second-generation anti-histamines, focusing on suppressive effect, rapidness of action, and impairment of task performance.

## 2. Materials and Methods

### 2.1. Subjects

Ten healthy volunteers (6 men and 4 women) aged 25 to 42 years (mean: 33.50) enrolled in this study. Subjects were excluded if they had taken any drug that had antihistamine action or any corticosteroid (oral/topical) within seven days prior to participation. The study was approved by the Medical Ethics Committee of Kurume University (Approval no.09028). Verbal and written information on the study was supplied, and all subjects gave written consent for study participation.

### 2.2. Study Design

A double-blinded, crossover, placebo-controlled protocol was used. 

Each subject was given one of four kinds of treatment. We selected 3 second-generation anti-histamines, olopatadine hydrochloride (olopatadine) 5 mg, bepotastine besilate (bepotastine) 10 mg, and fexofenadine hydrochloride (fexofenadine) 60 mg, which show relatively short time to maximum concentration (Tmax). These drugs and a placebo (pantethine) were placed in capsules for oral administration. Testing for each treatment was separated by a washout interval of at least 7 days. Subjects received active or placebo treatment at 9:00 a.m. Neither participants nor physicians had information on the drugs being tested.

### 2.3. Histamine-Induced Wheal/Flare Response

Histamine prepared as 0.1% solution was injected into the flexor surface of the forearms (alternating the right and left side) using an iontophoreser (UI-2060, BS Medical, Tokyo). The electrode had a tip diameter of 10.0 mm. Current was applied for 60 s at 0.1 mA. Fifteen minutes after iontophoresis, the wheal and flare areas were measured by “Image J” software and expressed as a percentage of the area observed at 0 hour for all time points. 

### 2.4. Subjective Assessment of Psychomotor Activity

The subjective itch intensity and sleepiness were assessed on a visual analogue scale (VAS) 10, 13, and 15 minutes after each iontophoresis, and the mean of the three scores was considered as itch score for that time point. On VAS of itch intensity, 0 represented “no itch sensation,” and 100 represented “unbearable itch.” On VAS of sleepiness, 0 represented “not sleepy,” and 100 represented “unbearably sleepy”.

### 2.5. Objective Assessment of Psychomotor Activity

Effects on objective cognitive function were assessed by typing speed and accuracy for one minute in triplicate at 0, 1, 2, 4 and 8 hours using the type training software “MIKA TYPE” [3]. The numbers of characters typed and errors made per minute were recorded in triplicate, and the mean values were considered as performance score for that time point.

### 2.6. Statistical Analysis

All data were expressed as mean ± SE. Statistical analysis of the difference between different treatment groups was performed by the Tukey's test (distribution was normal; distribution was tested by Kolmogorov-Smirnov test) with a significance level of 5% (Dr. SPSS II, SPSS Japan Inc., Tokyo). 

## 3. Results and Discussion

**Figure 1:**
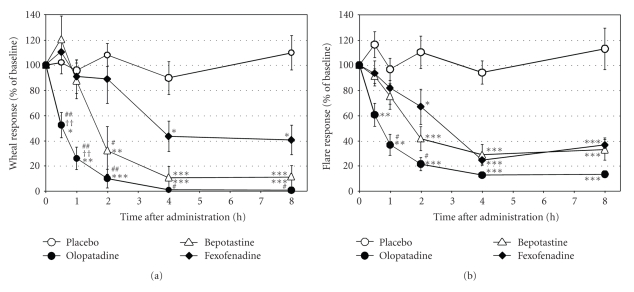
Inhibitory effects of anti-histamines on histamine-induced wheal response (a) and flare response (b) after histamine iontophoresis treatment (*n* = 10). Results are presented as mean ± SE. *P < .05, **P < .01, and ***P < .001 (olopatadine versus placebo); ^††^ *P *< .01 (olopatadine versus bepotastine); ^#^ *P *< .05, ^##^ *P *< 0.01 (olopatadine versus fexofenadine).

Histamine iontophoresis produced wheal and flare, which disappeared in about 40 minutes. The wheal and flare areas induced by the initial iontophoresis at 0 hour were 0.79 cm^2^ and 4.34 cm^2^ in placebo group, 1.04 cm^2^ and 5.13 cm^2^ in olopatadine group, 0.86 cm^2^ and 5.13 cm^2^ in bepotastine group, and 0.91 cm^2^ and 5.12 cm^2^ in fexofenadine group, respectively. All three drugs significantly suppressed the wheal response, compared to placebo (Figure 1(a)). Olopatadine was the only drug that showed a significant suppression of wheal response versus placebo at 0.5 hours and 1 hour and was significantly more effective than bepotastine and fexofenadine. The wheal response at 2 hours was significantly suppressed by olopatadine and bepotastine, compared to placebo and fexofenadine. After 4 hours, all 3 drugs significantly suppressed wheal response, compared to placebo although olopatadine was significantly superior to fexofenadine.

The 3 drugs were also effective in suppressing flare response, compared to placebo (Figure 1(b)). Similar to wheal response, olopatadine was the only drug that showed significant suppression of flare response versus placebo at 0.5 hours and 1 hour and was significantly more effective than fexofenadine at one hour and two hours. After two hours, all three drugs significantly suppressed flare response, compared to placebo. These findings indicated the difference in suppressing effect among the three drugs; olopatadine was the fastest and most potent medication, followed by bepotastine and fexofenadine. Pharmacokinetic properties of each drug may contribute to this difference. The rapidness of action is influenced by Tmax. Olopatadine has the shortest Tmax (1.0 hour), followed by bepotastine (1.2 hour) and fexofenadine (2.2 hours) [4–6]. For the three drugs studied, the rapidness of suppression of skin responses almost coincided with Tmax. 

**Figure 2:**
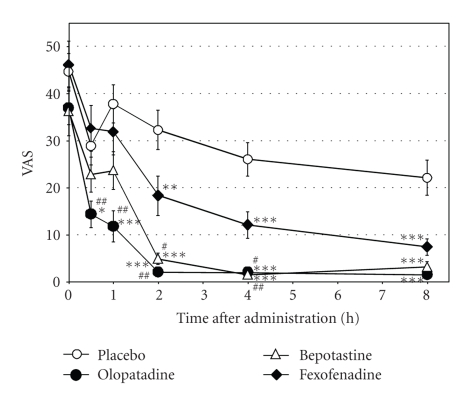
Inhibitory effects of anti-histamines on VAS values of histamine-induced itching sensation after histamine iontophoresis (*n* = 10). Results are presented as mean ± SE. *P < .05, **P < .01, and ***P < .001 (olopatadine versus placebo); ^#^ *P *< .05, ^##^ *P *< .01 (olopatadine versus fexofenadine).

The subjective itch intensity was assessed on a VAS. The itch score at 0 hour before medication was approximately 40 (36.2 to 46.1). The placebo group showed the highest score at 0 hour, which tended to gradually decrease. VAS scores at 0.5 hours and 1 hour were decreased only by olopatadine, and the decrease was significant, compared not only to placebo but also to fexofenadine (Figure 2). After two hours, all three drugs significantly decreased itch scores versus placebo. Decreases seen in olopatadine and bepotastine at two hours and four hours were significantly higher, when compared to fexofenadine. The significantly higher suppression of skin response and itch by olopatadine was consistent with the results from previous histamine iontophoresis studies [7, 8]. Olopatadine exhibited a potent noncompetitive antagonism for the human H_1_ receptor, which may contribute to the activity of this drug [9].

**Figure 3:**
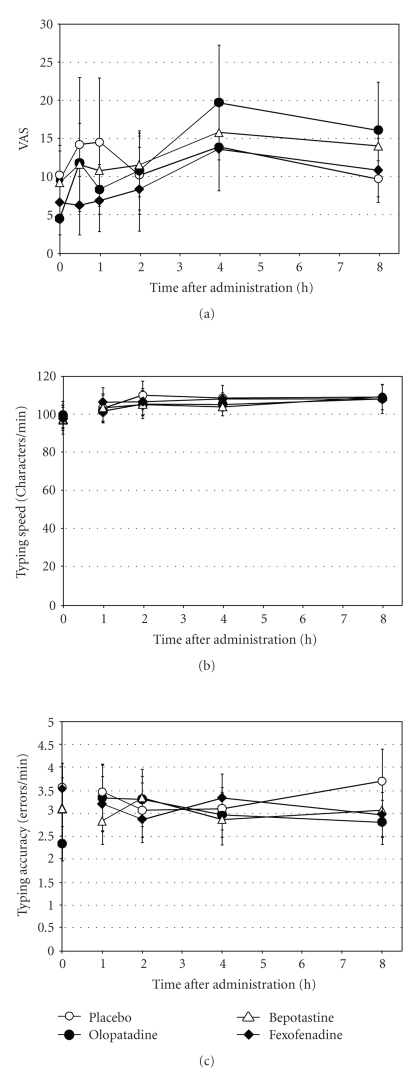
Evaluation of sedative effects by VAS score for sleepiness (a), typing speed (b), and typing accuracy (c) (*n* = 10). (a) VAS scores: 0 (no drowsiness) to 100 (extensive drowsiness). Resutls are presented as mean ± SE.

Subjective sleepiness was assessed on a VAS. No significant differences were observed for sleepiness up to 8 hours between the drug-treated groups and placebo group (Figure 3(a)). However, VAS scores varied very much among subjects. In fact, some subjects, though small in number, complained of profound sleepiness with every drug, while other subjects responded differently to the three drugs. This finding supported a large individual differences in drug-induced sleepiness, reported by previous publications.

Effects on objective cognitive function were assessed by typing speed and accuracy. No significant differences were noted in typing speed (characters typed per minute) or accuracy (errors made per minute) between drug-treated groups and placebo group at any time points between one hour and eight hours (Figures 3(b) and 3(c)). Second-generation anti-histamines have lower central nervous system penetrance [10], resulting in reduced central nervous system effects, compared to the first-generation anti-histamines. This may explain the lack of performance impairment, an objective indicator of sedation, in our study. 

Evaluation of three second-generation anti-histamines by means of histamine iontophoresis showed that they were similar in terms of central nervous system side effects but were different in terms of rapidness of action and efficacy. Olopatadine was found to be the fastest and most potent drug. When a rash recurs after remission of acute or chronic urticaria, an anti-histamine is prescribed for prompt relief of symptoms. Rapid improvement of the symptoms not only provides therapeutic benefits to patients but also improves quality of life by increasing patient's confidence in alleviating their anxiety. The data on rapidness of action seen in this study provide important information that will help us to choose a suitable anti-histamine for individual urticaria patients.
